# Myosin 1b and F-actin are involved in the control of secretory granule biogenesis

**DOI:** 10.1038/s41598-017-05617-1

**Published:** 2017-07-12

**Authors:** Charlène Delestre-Delacour, Ophélie Carmon, Fanny Laguerre, Catherine Estay-Ahumada, Maïté Courel, Salah Elias, Lydie Jeandel, Margarita Villar Rayo, Juan R. Peinado, Lucie Sengmanivong, Stéphane Gasman, Evelyne Coudrier, Youssef Anouar, Maité Montero-Hadjadje

**Affiliations:** 1 Normandie Univ, UNIROUEN, INSERM, U1239, Laboratoire de Différenciation et Communication Neuronale et Neuroendocrine, Institut de Recherche et d’Innovation Biomédicale de Normandie, 76000 Rouen, France; 2 Université de Strasbourg, CNRS UPR 3212, Institut des Neurosciences Cellulaires et Intégratives, 67000 Strasbourg, France; 3 Instituto de Investigación en Recursos Cinegéticos, Proteomics Core Facility, 13071 Ciudad Real, Spain; 4 Laboratory of oxidative stress and neurodegeneration, Facultad de Medicina de Ciudad Real, 13071 Ciudad Real, Spain; 5 Institut Curie - PSL Research University, Membrane Dynamics and Mechanics of Intracellular Signaling Laboratory, Nikon Imaging Centre, 75005 Paris, France; 6 CNRS UMR 144 Cell Signaling and Morphogenesis, Institut Curie, 75005 Paris, France; 7 CNRS-UPMC FRE3402, Pierre et Marie Curie University, 75252 Paris, Cedex 05 France; 8 University of Oxford, Sir William Dunn School of Pathology, Oxford, Oxfordshire United Kingdom

## Abstract

Hormone secretion relies on secretory granules which store hormones in endocrine cells and release them upon cell stimulation. The molecular events leading to hormone sorting and secretory granule formation at the level of the TGN are still elusive. Our proteomic analysis of purified whole secretory granules or secretory granule membranes uncovered their association with the actomyosin components myosin 1b, actin and the actin nucleation complex Arp2/3. We found that myosin 1b controls the formation of secretory granules and the associated regulated secretion in both neuroendocrine cells and chromogranin A-expressing COS7 cells used as a simplified model of induced secretion. We show that F-actin is also involved in secretory granule biogenesis and that myosin 1b cooperates with Arp2/3 to recruit F-actin to the Golgi region where secretory granules bud. These results provide the first evidence that components of the actomyosin complex promote the biogenesis of secretory granules and thereby regulate hormone sorting and secretion.

## Introduction

Besides the constitutive secretory pathway which is involved in the renewing of plasma membrane and extracellular matrix in all eukaryotic cell types, a regulated secretory pathway is specialized in hormone release in endocrine cells. The vesicular membrane structures at the origin of these secretory pathways, called constitutive vesicles and secretory granules respectively, arise by budding from the trans-Golgi network (TGN) membrane. However, the molecular mechanisms linking hormone sorting, TGN membrane and secretory granule formation are still poorly understood. Like all biological membranes, the TGN membrane is composed of a specific lipid and protein mix resulting in a proper lateral organization that supports the function of the TGN compartment^1^. Membrane-interacting cytosolic proteins are necessary to the dynamic morphology and to the functional organization of the TGN membrane, and include for example enzymes involved in the phospholipid remodeling^2^ or proteins with Bin/Amphiphysin/Rvs domains capable of sensing and/or stabilizing membrane curvature^3, 4^. Actin and its associated motors have also been shown to interact with the TGN membrane and to modulate its topology, as demonstrated for myosin II which promotes the fission of constitutive secretory vesicles^5^, and myosin 1b which induces the formation of post-Golgi carriers in HeLa cells^6^. Interestingly, proteomic studies of secretory granules identified many actin-interacting proteins, including myosins^7, 8^, which could contribute to the control of different steps of endocrine secretion. Among these, myosin VI has been shown to control secretory granule exocytosis^9^ whereas myosin 1b has currently no known function in endocrine cells.

Since myosin 1b binds to F-actin through its motor domain and to membrane phosphoinositides probably through its pleckstrin homology motif^10, 11^ on the one hand, and on the other, facilitates the extraction of tubular structures under conditions of increasing membrane extension^12^, we postulated that this myosin and associated F-actin are good candidates to regulate the early steps of secretory granule formation in endocrine cells. In the present study, we observed the occurrence of myosin 1b (Myo1b) in the TGN area and on immature secretory granules of endocrine cells, and found that depletion of Myo1b using small interfering RNA (siRNA) significantly reduces *(i)* the number of secretory granules, *(ii)* regulated secretion and *(iii)* the distribution of F-actin in the Golgi region. In fact, F-actin depolymerization and Arp2/3 complex inhibition phenocopied the effect of Myo1b down-regulation on secretory granule formation. Collectively these results show for the first time the implication of the actomyosin system in the biogenesis of secretory granules and thus in hormone sorting through the regulated secretory pathway in endocrine cells.

## Results

### Myosin 1b is associated with the trans-Golgi network and immature secretory granules in neuroendocrine PC12 cells

**Figure 1:**
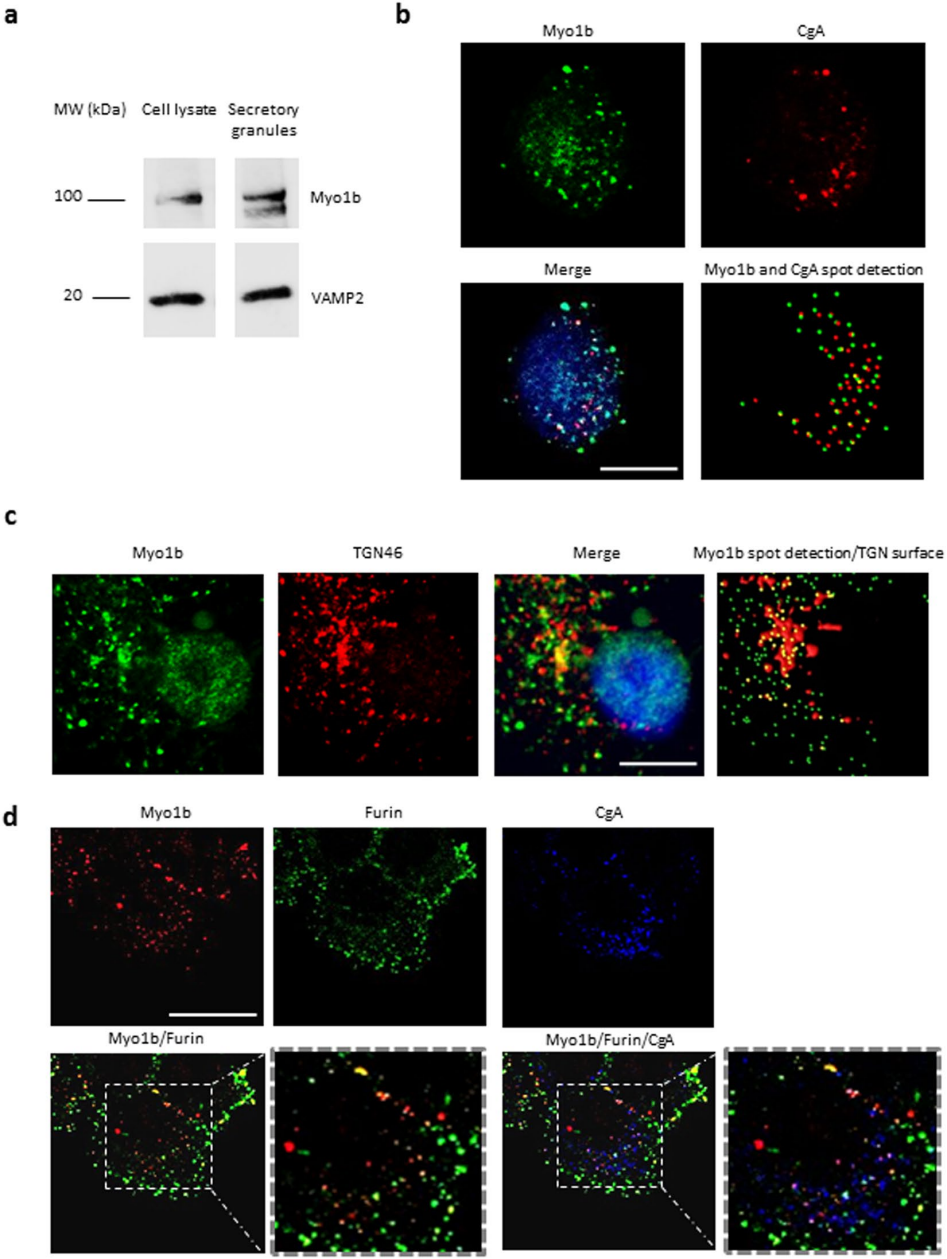
Myosin 1b is associated with the trans-Golgi network and secretory granules in PC12 cells. (**a**) Cropped and color inverted blots showing protein expression levels of myosin 1b (Myo1b) and VAMP2 in a PC12 cell lysate and secretory granule-containing fraction. (Full image of each tested protein are reported in Supplementary Figure S1). (**b**–**d**) PC12 cells were immunolabeled with anti-TGN46, Myo1b, CgA and furin antibodies. (**b**) Representative confocal microscopy sections throughout the cell show a partial overlap of Myo1b and CgA-containing secretory granules (47 ± 18%, with a 0.409 Pearson correlation coefficient, from three independent experiments, n = 39 cells). (**c**) Representative confocal microscopy sections throughout the cell show an overlap of Myo1b labeling and a TGN marker. (**d**) Representative confocal microscopy sections throughout the cell show an overlap of Myo1b and CgA-containing immature secretory granules labeled by furin (89 ± 8%, from three independent experiments, n = 66 cells). Scale bar represents 10 µm.

We first analyzed the expression and distribution of myosin 1b (Myo1b) in neuroendocrine PC12 cells. Western blot analysis of PC12 cell lysates and purified secretory granules revealed the cofractionation of Myo1b and VAMP2 (vesicle-associated membrane protein 2), a specific marker of secretory granule membrane (Fig. 1a). Analysis of Myo1b distribution in PC12 cells by confocal microscopy coupled to immunofluorescence (IF) revealed that this protein is associated with 47 + 18% of secretory granules labeled with chromogranin A (CgA), a marker of secretory granules (Fig. 1b). Using antibodies raised against TGN46, a marker of the trans-Golgi network, and against furin, a prohormone convertase mainly localized in immature secretory granules just after their budding from the TGN membrane, we observed that Myo1b is mainly located in the TGN area (Fig. 1c) and in 89 + 8% of immature CgA-containing secretory granules (Fig. 1d). Together, these results show that Myo1b is associated with secretory granules at the level of the TGN, most likely to promote the budding of immature secretory granules.

### Myosin 1b controls the biogenesis of secretory granules

**Figure 2:**
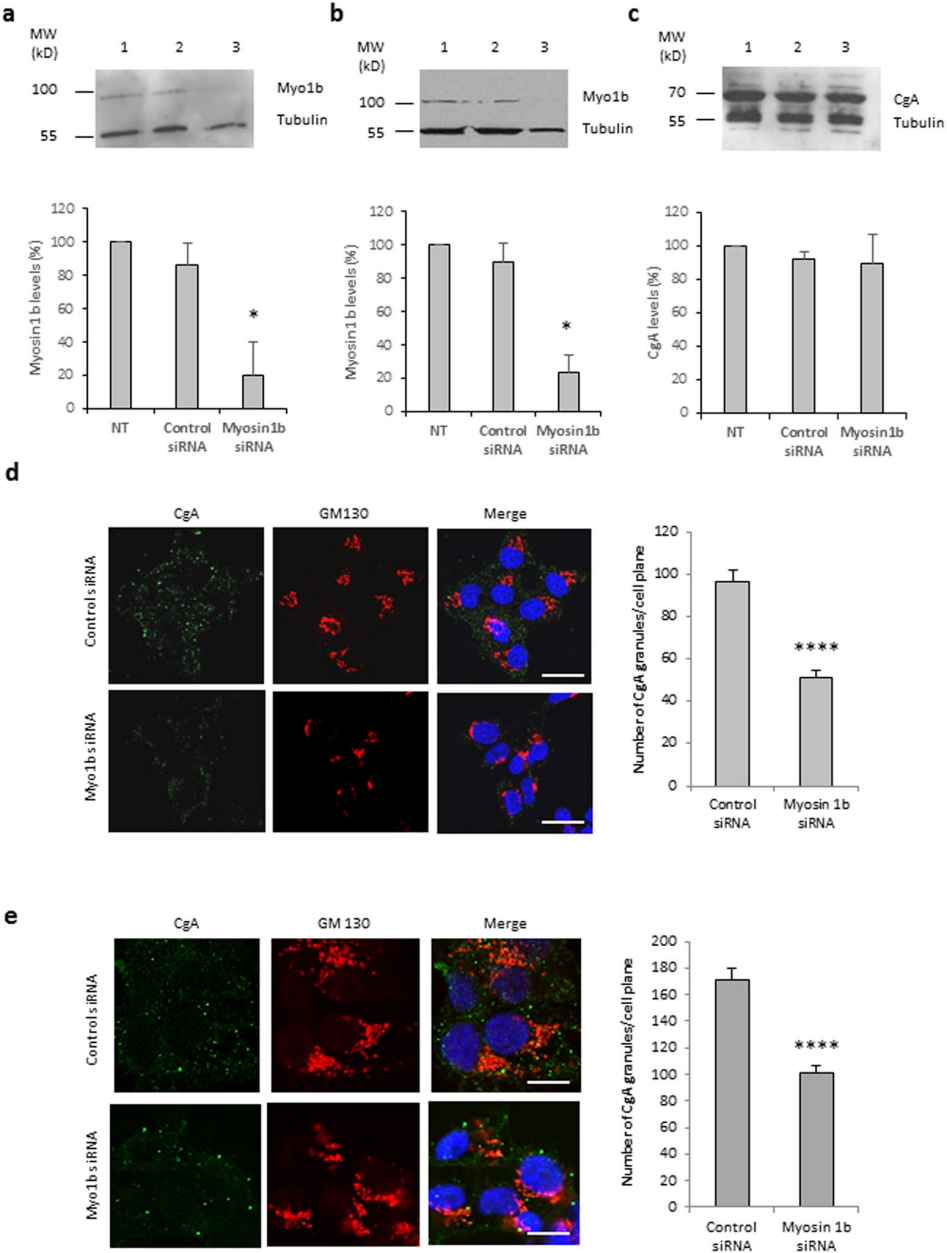
Myosin 1b depletion reduces the biogenesis of CgA-containing granules in PC12 and COS7 cells. (**a**–**c**) Protein extracts were analyzed by immunoblotting. Cropped blots showing protein expression levels of Myo1b and tubulin in lysates of COS7-CgA cells (**a**) or PC12 cells (**b**), non-transfected (lane 1), transfected with control siRNA (lane 2) or myosin 1b siRNA (lane 3). (**c**) Cropped blots showing protein expression levels of CgA and tubulin in lysates of PC12 cells, non-transfected (lane 1), transfected with control siRNA (lane 2) or myosin 1b siRNA (lane 3). Histograms represent a semi-quantitative analysis of the amount of myosin 1b or CgA found in cells transfected with control siRNA or myosin 1b siRNA from three independent experiments. *P < 0.05 (Mann-Whitney test). (Full-length blots of each tested protein are reported in Supplementary Figure S2). (**d**) PC12 cells were transfected with control siRNA or Myo1b siRNA, fixed, immunolabelled with anti-CgA and GM130 antibodies, and analyzed by confocal microscopy. Representative confocal microscopy sections throughout the cells are shown. The scale bar represents 10 µm. The number of CgA granules in PC12 cells transfected with control siRNA or Myo1b siRNA was quantified and expressed as mean + s.e.m. from three independent experiments (n = 36 cells). ****P < 0.0001 (Student’s t -test with Welch’s correction). (**e**) COS7-CgA cells were transfected with control siRNA or Myo1b siRNA, fixed, immunolabelled with anti-CgA and GM130 antibodies, and the distribution of CgA was analyzed by confocal microscopy. Representative confocal microscopy sections throughout the cells are shown. The scale bar represents 10 µm. The number of CgA granules in COS7-CgA cells, transfected with control siRNA or myosin 1b siRNA, was quantified and expressed as mean + s.e.m. from three independent experiments (n = 43 cells). ****P < 0.0001 (Mann-Whitney test).

**Figure 3:**
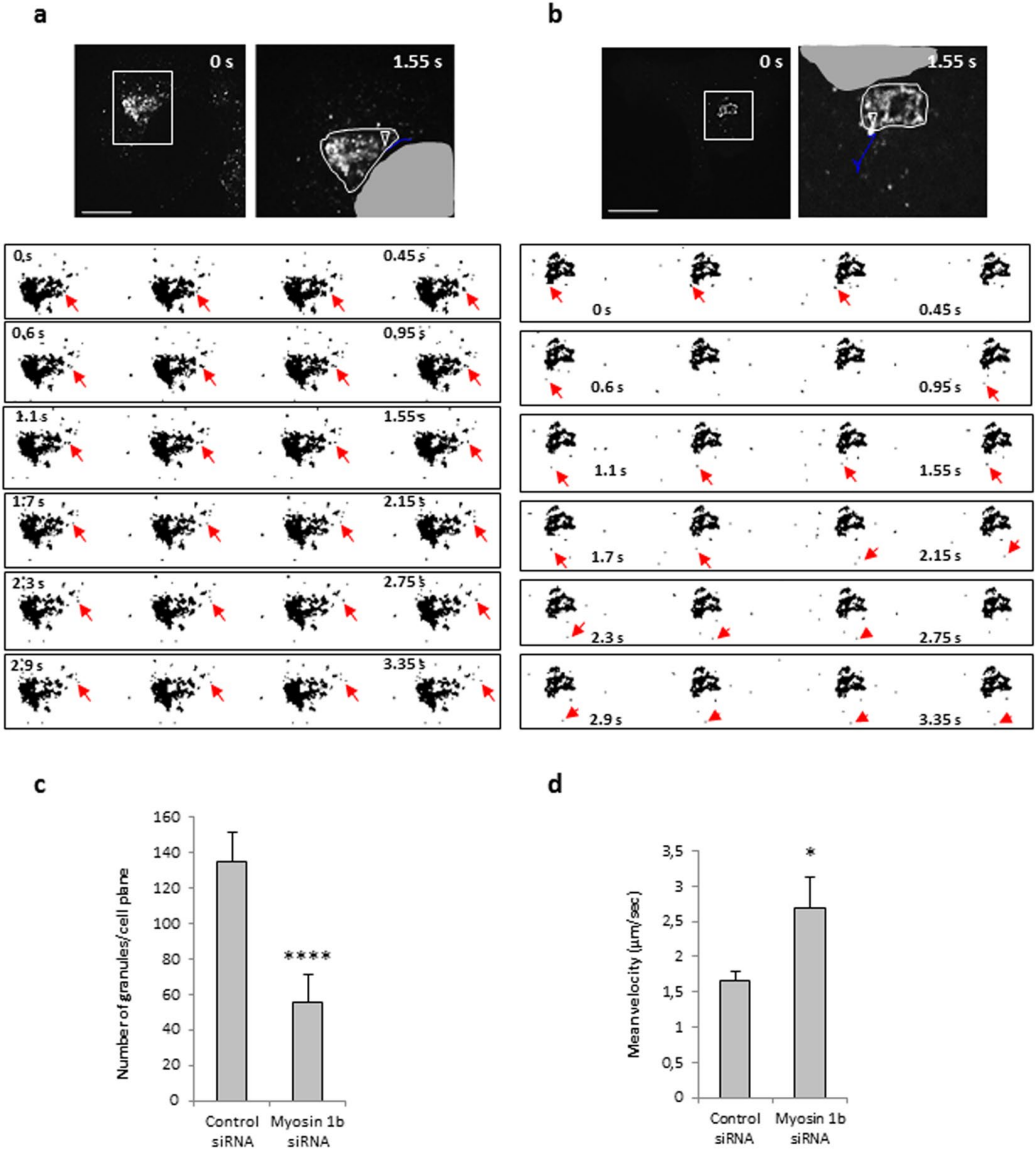
Myo1b depletion alters the number and the velocity of CgA granules. COS7 cells were first transfected with control siRNA (**a**) or myosin1b siRNA (**b**), and 24 h later with a plasmid encoding CgA-GFP. CgA-GFP carriers were monitored 5 h later at 37 °C by time-lapse imaging using spinning-disc confocal microscopy (see Movies 1 and 2). The first frames of representative movies (that cover 3.5 s) revealing the sequence of events of granule formation are shown. White boxes indicate the enlarged TGN region shown in image sequences. The right image in each panel corresponds to the enlarged region showing granule trajectory (in blue) along the image sequence, using the ‘manual tracking’ plugin of Image J. The arrow head indicates the position of the granule at the beginning of the tracking. The white line delimitates the Golgi region and the grey zone corresponds to part of the nucleus. The scale bars represent 10 µm. The red arrows indicate the position of the tracked granule every 150 ms. (**c**) The number of CgA-GFP granules was quantified, and expressed as mean + s.e.m. from three independent experiments (n = 20–24 cells). ****P < 0.0001 (Mann-Whitney test). (**d**) The mean velocities were calculated as the cumulated displacement of the granule from the location of its first observation to the end of the observation period with respect to the time of observation, and expressed as mean + s.e.m. from three independent experiments (N = 14–19 cells, n = 22–69 granules). *P < 0.05 (Mann-Whitney test).

To demonstrate the implication of Myo1b in the biogenesis of secretory granules, we used the neuroendocrine PC12 cells as well as a simplified model consisting of COS7 cells expressing CgA which has been shown to induce secretory granule-like structures in these cells^8, 13^. Myosin 1b gene silencing in these cells was achieved through a home-designed short interfering RNA (Myo1bH siRNA) validated in the study of Almeida *et al*.^6^, which affords a strong reduction in Myo1b expression. Indeed, transfection of this siRNA significantly reduced Myo1b expression in both COS7-CgA (Fig. 2a) and PC12 (Fig. 2b) cells. This decrease in Myo1b expression did not alter CgA expression in PC12 cells (Fig. 2c), thus ruling out the possibility of an effect of Myo1b through altered CgA expression. Under these conditions and using confocal microscopy coupled to IF, we observed a significant reduction in the number of CgA-containing granules in both PC12 (Fig. 2d) and COS7-CgA (Fig. 2e) cells. This result shows that Myo1b could play a key role in the formation of secretory granules. We then investigated whether Myo1b knockdown alters the formation of secretory granules at the TGN level by fast time-lapse imaging of COS7 cells expressing CgA-GFP, using a spinning disc-equipped confocal microscope. Five hours after cell transfection, we observed numerous CgA-GFP-containing granules around the Golgi area moving towards the cytoplasm in control siRNA-treated cells (Fig. 3a; Movie 1), and we found that the number of CgA-GFP granules generated was significantly reduced in Myo1b siRNA-treated cells (Fig. 3b,c; Movie 2). Moreover, in the latter case, the granules generated moved faster than those observed in control cells treated with a scramble siRNA (Fig. 3d; Movies 1, 2). Thus, the absence of Myo1b alters not only the number but also the dynamics of CgA-GFP containing vesicles, which suggests together with the distribution data described above that the motor protein is involved in the initiation of secretory granule formation from the Golgi complex.

### Myosin 1b depletion increases basal secretion

**Figure 4:**
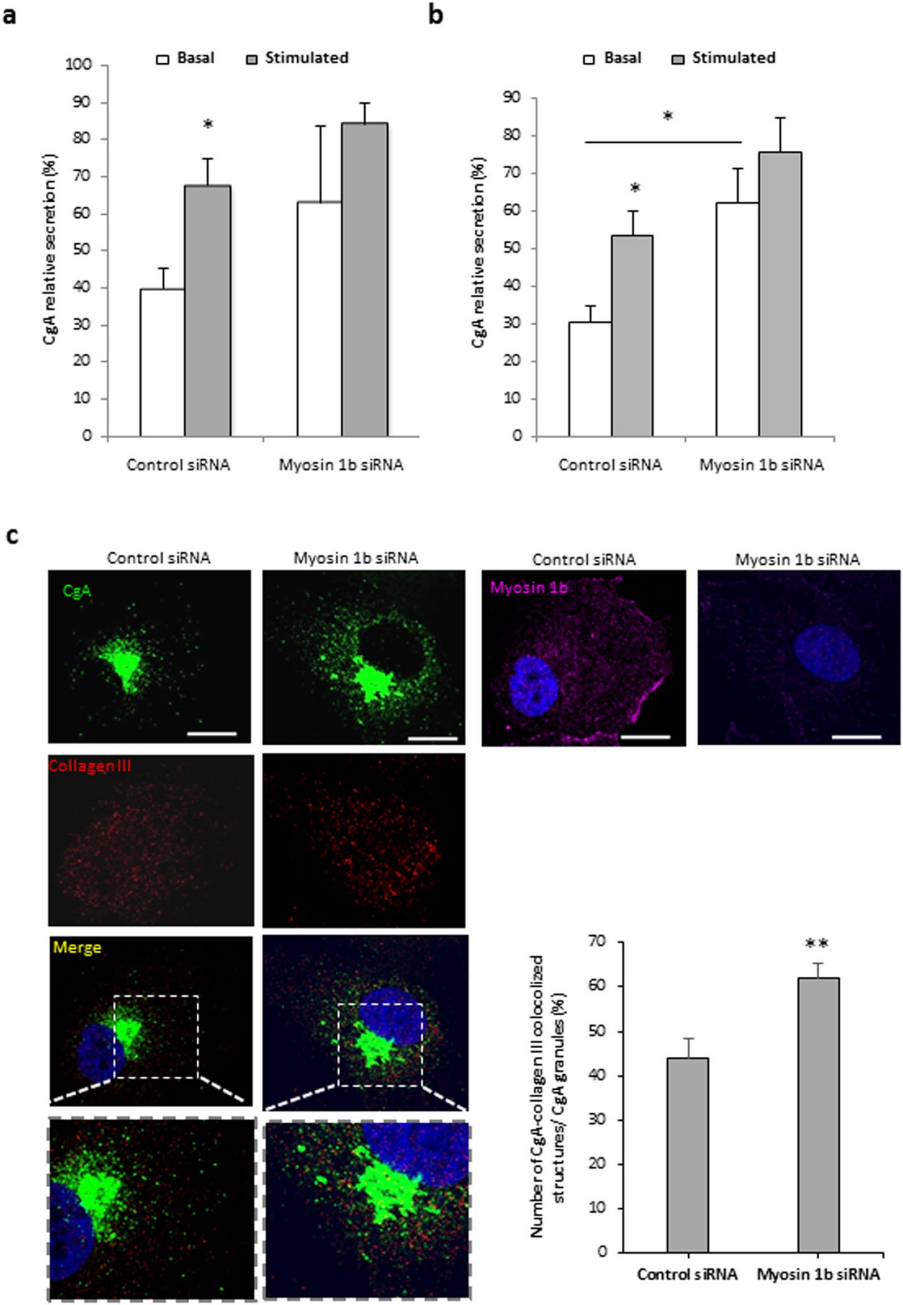
Myosin 1b depletion leads to CgA leakage through the constitutive secretory pathway. Quantification after Western blot analysis of CgA secretion and content from COS7-CgA cells (**a**) and PC12 cells (**b**), transfected with control or myo1b siRNA, in basal or 2 mM Ba^2+^-stimulated conditions. Normalized CgA release in the medium relative to total CgA (medium + cell content) is determined. The values represent the means ± s.e.m. from four independent experiments. *P < 0.05 (Mann-Whitney test). (**c**) COS7 cells were transfected with control siRNA or Myo1b siRNA and with a plasmid encoding CgA-GFP, fixed, immunolabeled with anti-collagen III or Myo1b antibody, and the distribution of CgA was analyzed by confocal microscopy. The scale bar represents 10 µm. The number of CgA-GFP/collagen III colocalized structures in COS7-CgA cells, transfected with control or myosin 1b siRNAs, was quantified and expressed as percentages of CgA/collagen III colocalized structures relative to total CgA granules. The values represent the means ± s.e.m. from three independent experiments (n = 45 cells). **P < 0.01 (Student’s t-test with Welch’s correction).

Given the impact of Myo1b expression on the number of secretory granules, we analyzed the effect of this myosin on the secretory competence of PC12 and COS7-CgA cells using CgA release as an index of the regulated secretory pathway. This analysis showed that Myo1b knockdown results in increased basal release of CgA in both cell models (Fig. 4a,b). We also studied the distribution of CgA in the constitutive and the regulated secretory pathways in the absence of Myo1b. Using an antibody raised against collagen III, a protein of the extracellular matrix of fibroblasts which is addressed to the constitutive secretory pathway, we found that Myo1b down-regulation provokes a significant increase in the number of vesicular organelles containing both collagen III and CgA (Fig. 4c). These results indicate that CgA is misrouted to the constitutive secretory pathway in the absence of Myo1b, leading to its increased release in basal conditions. Together, these data show that Myo1b controls secretory protein sorting toward the regulated pathway.

### Actin and actin-related proteins are also required for the biogenesis of secretory granules

**Figure 5:**
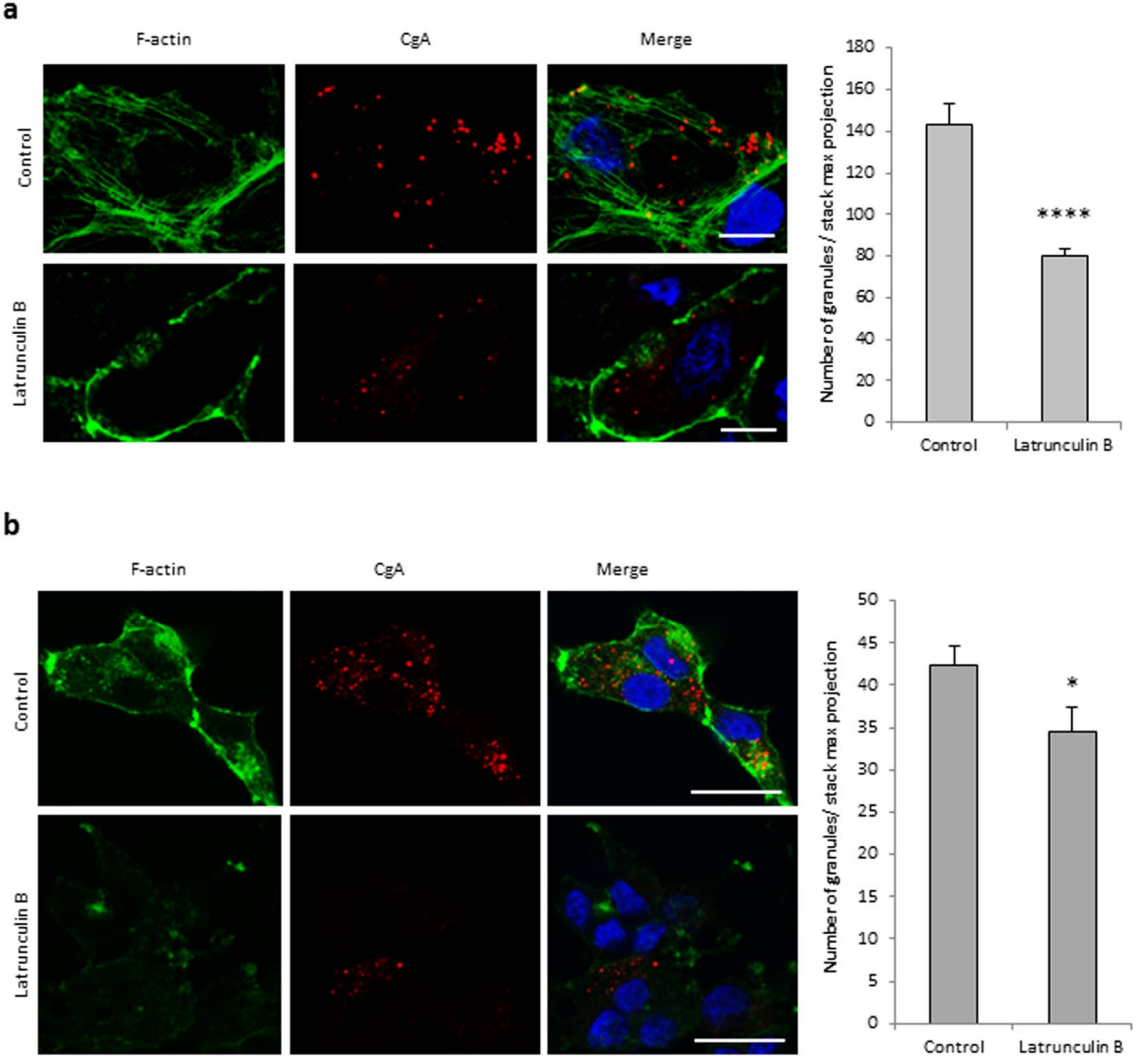
F-actin is required for the biogenesis of secretory granules. COS7-CgA cells (**a**) and PC12 cells (**b**) were treated using DMEM alone (control) or supplemented with latrunculin B. They were labeled with fluorescent phalloidin (F-actin), immunolabeled with anti-CgA antibody and analyzed by confocal microscopy. Projected images of 26 0.3 µm-thick confocal sections throughout the cells are shown. The scale bar represents 20 µm. The number of CgA granules in control and treated cells was quantified and expressed as mean + s.e.m. from three independent experiments (n = 30 cells). *P < 0.05, ****P < 0.0001 (Mann-Whitney test).

**Figure 6:**
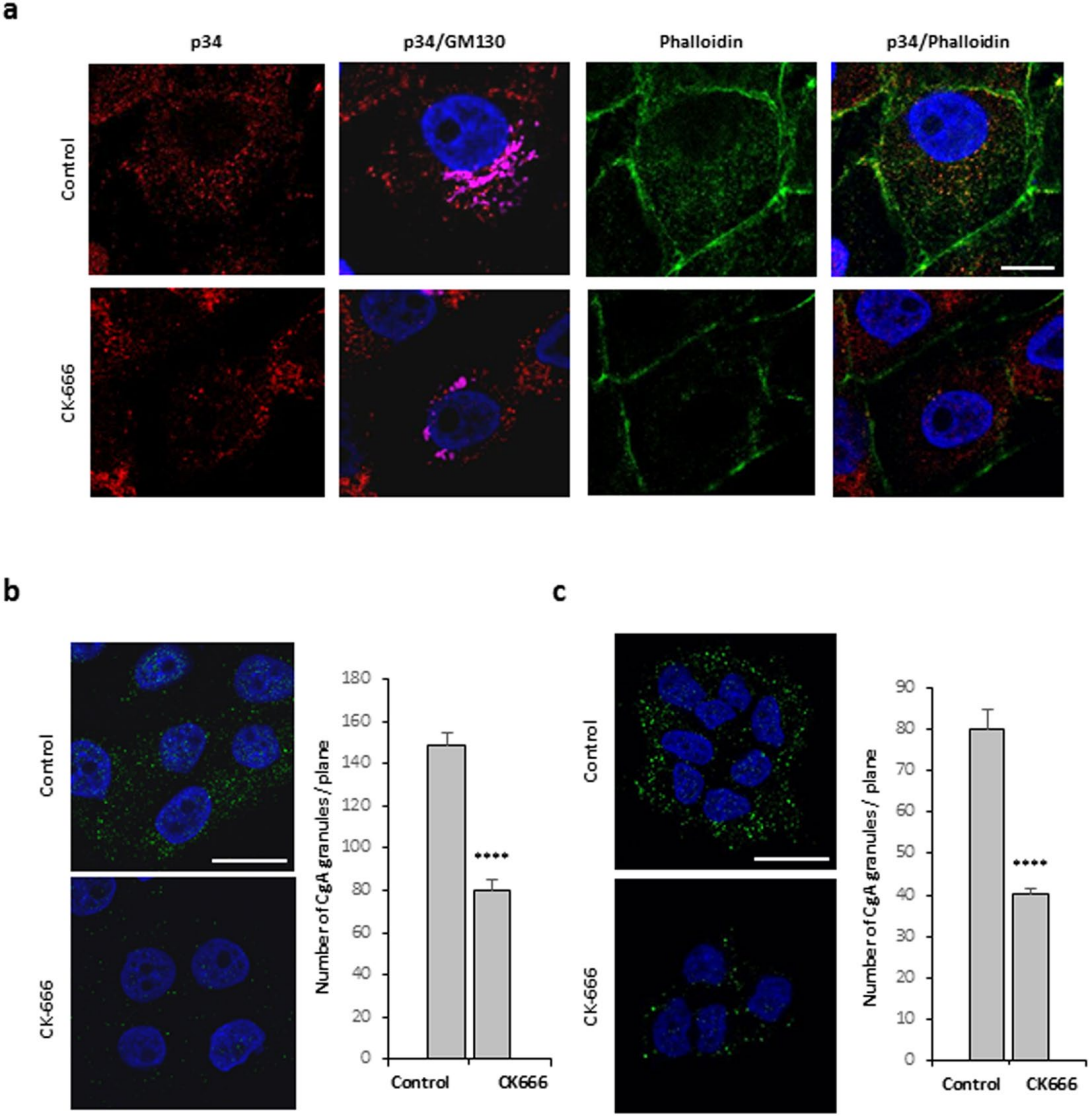
Arp2/3 complex activation is required for the biogenesis of secretory granules. (**a**) COS7-CgA cells were treated with DMSO alone (control) or with the Arp2/3 inhibitor (CK-666). The distribution of the Arp2/3 complex and F-actin was assessed by immunofluorescence with anti-p34 antibody and by fluorescent phalloidin, respectively, and was analyzed by confocal microscopy. A single focal medial plane of GM130 (magenta), p34 (red) and F-actin (green) distribution is shown. Scale bar represents 20 μm. COS7-CgA cells (**b**) and PC12 cells (**c**) were treated with DMSO alone (control) or with the Arp2/3 inhibitor (CK-666). They were immunolabeled with anti-CgA antibody and analyzed by confocal microscopy. Representative confocal microscopy sections throughout the nucleus are shown. The scale bar represents 20 µm. The number of CgA granules in cells treated with DMSO alone (control) or with the Arp2/3 inhibitor (CK-666) was quantified and expressed as mean + s.e.m. from three independent experiments (n = 40 cells). ****P < 0.0001 (Student’s t -test with Welch’s correction).

In addition to those previously identified through proteomic characterization of whole secretory granules^8^, further analysis of purified secretory granule membranes revealed the occurrence of several other F-actin components, effectors and regulators with known functions at the level of the Golgi complex (Table 1). Among these proteins, Arp2/3 and actin cytoplasmic 1 and 2 have been reported to promote the formation of post-Golgi carriers by regulating membrane remodeling at the TGN in HeLa cells^6^, suggesting that actin could cooperate with Myo1b at the level of the TGN membrane to regulate secretory granule formation. To determine the role of F-actin in secretory granules biogenesis, we depolymerized F-actin using latrunculin B and inhibited actin nucleation mediated by the Arp2/3 complex using CK-666, in COS7-CgA and PC12 cells. F-actin depolymerization led to a significant decrease in the number of CgA granules (up to 50% and 10% in COS7-CgA and PC12 cells, respectively) (Fig. 5a,b), thus revealing a key function of F-actin in the regulation of secretory granule formation. IF analysis of the Arp2/3 p34 subunit distribution confirmed that CK-666 treatment inhibits the recruitment of Arp2/3 complex and F-actin to the Golgi region (Fig. 6a). Inhibition of Arp2/3 complex also provoked a significant reduction in the number of secretory granules (up to 50% in COS7-CgA and PC12 cells) (Fig. 6b,c). Taken together, these observations demonstrate that Arp2/3-dependent recruitment of F-actin in the Golgi area is required for the regulation of secretory granule biogenesis.

**Table 1:** F-actin components, effectors and regulators, with known functions at the Golgi complex, identified in the membrane fraction of purified granules from COS7-CgA cells.

Name	Access number	Function	References
Actin cytoplasmic 1	P60709	Golgi-derived transport carrier biogenesis and Golgi-to-ER protein transport	25
Actin cytoplasmic 2	P63261		26
Actin related protein 2/3 complex subunit 2	O15144	Associated to actin filaments and involved in TGN budding	6
		TGN budding	27
Cofilin-1	P23528	Signalling pathway that regulates actin assembly in neuronal Golgi membranes.	
Profilin	P35080		28
Secretory carrier membrane protein 3	O14828	Golgi-derived transport carrier biogenesis and Golgi-to-ER protein transport	29

Identification was based on human sequences, as well as those of other mammalian species. Only proteins that were identified with one or more high scoring peptides from Mascot were considered to be true matches. “High scoring peptides” corresponded to peptides that were above the threshold in Mascot (P < 0.05) searches. The table shows a non-redundant list of the proteins and the reference to the Uniprot-access number used in database searches (http://uniprot.org).

### Myosin 1b promotes the recruitment of Arp2/3-dependent F-actin to the Golgi area

**Figure 7:**
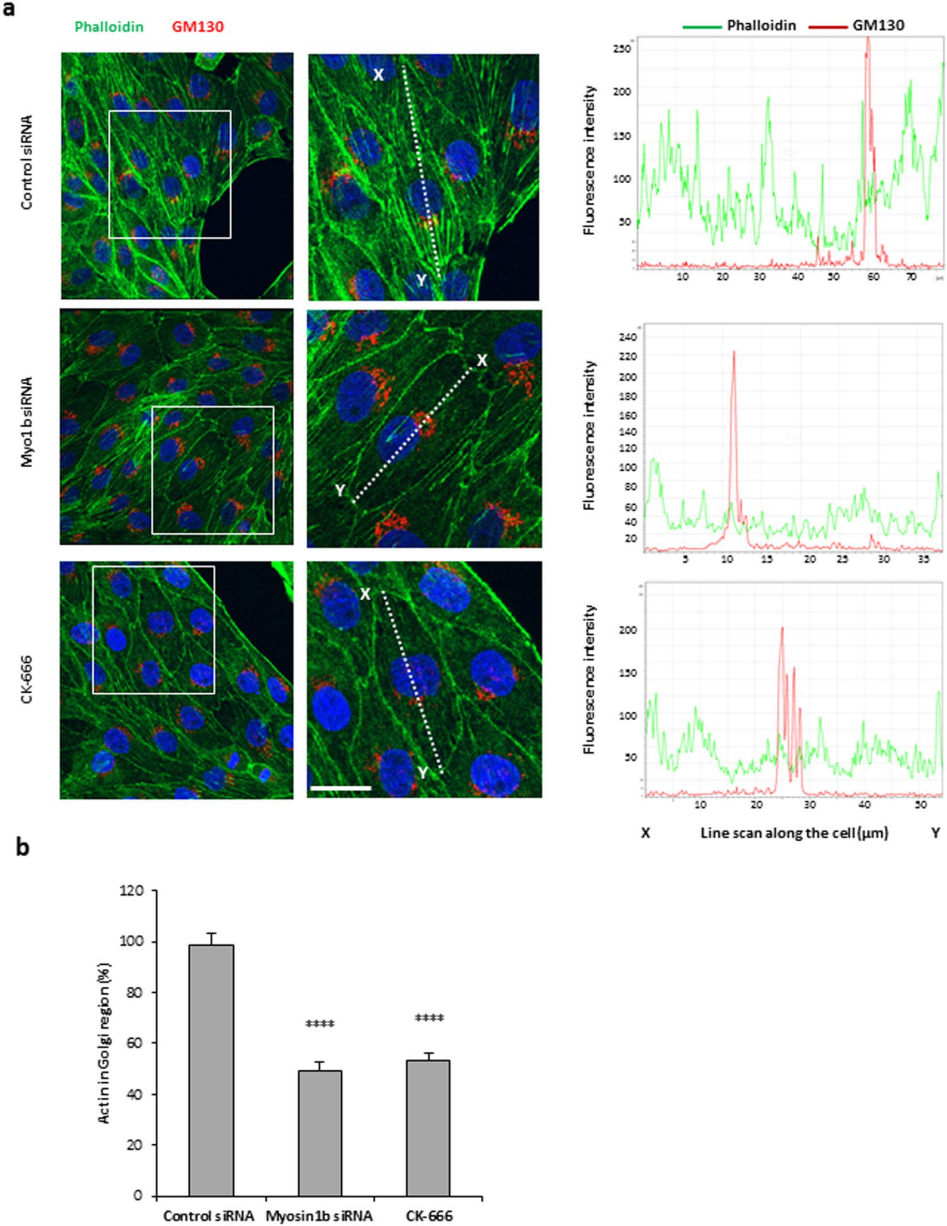
Myosin 1b knockdown and depletion of the Arp2/3 complex reduce F-actin distribution in the Golgi area. (**a**) COS7-CgA cells were either transfected with control siRNA or Myo1b siRNA, or treated with the Arp2/3 inhibitor CK-666. F-actin and the Golgi complex were labeled with fluorescent phalloidin and immunolabelled with GM130 antibody, respectively, and cells were analyzed by confocal microscopy. Projected images of 20 0.3 µm-thick confocal sections throughout the cells are shown. The scale bar represents 20 µm. The fluorescence intensities along the white lines indicated in the merged pictures were quantified using the line scan mode of Leica software. Line scans taken through the cell are plotted in graphs in the right side of the corresponding images. (**b**) The localization of F-actin in the Golgi region was quantified and expressed as mean + s.e.m. from four independent experiments (n = 22 cells). ****P < 0.0001 (Student’s t -test with Welch’s correction).

Myo1b has the ability to interact with actin *via* its motor domain and with the membrane *via* its pleckstrin homology motif^11^. Using Myo1b siRNA and the Arp2/3 inhibitor CK-666, we investigated the impact of Myo1b and Arp2/3 complex on F-actin distribution in the Golgi area of COS7-CgA cells. Fluorescent phalloidin and GM130 antibody allowed the analysis of actin distribution around the Golgi area. Analysis of line scans of fluorescence intensity taken through the cell showed that Myo1b depletion and Arp2/3 inhibition lead to F-actin decrease in the Golgi area (Fig. 7a). Quantification of actin and Golgi complex colocalization showed that both Myo1b knockdown and Arp2/3 complex inhibition lead to a 50% decrease in actin occurrence at the Golgi complex (Fig. 7b). These results show that blockade of Arp2/3 complex phenocopies Myo1b knockdown and that both Myo1b and Arp2/3 complex are involved in the recruitment of F-actin to the Golgi area in secretory cells.

## Discussion

Secretory granules are key organelles allowing regulated hormone release in endocrine cells. Although considerable efforts have been devoted to understand the molecular events underlying secretory granule formation, the complex mechanisms that allow the specific sorting of hormones and the budding of secretory granules remain incompletely understood. It has been proposed that the sorting of peptide hormones through the regulated secretory pathway is controlled by chromogranins, which aggregate with peptides in the TGN lumen and function as the core around which budding of secretory granules is initiated from the TGN membrane^14^. In a previous study, we used a simplified model of CgA-induced vesicles in COS7 cells to identify potential effectors of granulogenesis through proteomic analysis of the newly formed granules. This analysis allowed the identification of a panel of cytosolic proteins interacting with actin, including myosins^8^, some of which have been associated with vesicular formation and exocytosis. Indeed, we identified myosin VI which is known to be involved in the regulated exocytosis of neuroendocrine cells^9, 15, 16^ and in the maintenance of the Golgi morphology^17^. We also identified Myo1b, which has been shown to have an anchoring role that allows TGN membrane deformation through its attachment to the actin cytoskeleton, leading to the biogenesis of post-Golgi carriers in HeLa cells^6^. A more detailed analysis by mass spectrometry of proteins associated with the membrane of newly formed granules allowed us to identify Arp2/3 complex and actin cytoplasmic 1 and 2, which could act in concert with myosins to regulate the formation of secretory granules in endocrine cells. Although several studies reported the implication of myosins and F-actin in the trafficking and exocytosis of secretory granules^18^, the role of these multi-functional proteins in the formation of secretory granules has never been reported. We found that Myo1b is associated with the TGN and immature secretory granules in PC12 cells, indicating that Myo1b could affect the early stages of secretory granule biogenesis by inducing the budding of immature vesicles from the TGN membrane. When Myo1b expression is knocked down, the number of secretory granules is significantly reduced, thus pointing out a possible direct role of this protein in the formation of secretory granules. In addition, time-lapse imaging revealed that Myo1b depletion impacts the dynamics of the formed granules. Since our previous studies showed that constitutive vesicles move faster than CgA-induced granules in COS7 cells^8^, our present observations suggest that the absence of Myo1b leads to misrouting of CgA toward the constitutive secretory pathway. In support of this finding, we observed higher basal secretion and a higher number of CgA-containing constitutive vesicles when Myo1b was knocked down. Taken together, these data strongly support a role of Myo1b in the biogenesis of functional secretory granules underlying hormone regulated release in endocrine cells.

In addition to myosins, we found that Arp2/3 complex and F-actin are associated to secretory granule membranes. But more than controlling the integrity of the Golgi complex, F-actin, together with Arp2/3, has been shown to mediate the effect of Myo1b on TGN membrane deformation in HeLa cells, as Myo1b actively tethers and orients polymerizing F-actin to generate the required mechanical force^6^. In the present study, we demonstrate for the first time that F-actin controls the biogenesis of secretory granules, most likely *via* its recruitment to the Golgi region through Arp2/3 activation. Indeed, the Arp2/3 complex binds to actin and was shown here to exert an active role in secretory granule formation, potentially by providing *de novo* actin tracks for membrane curvature, as demonstrated during secretory granule exocytosis^19, 20^. Therefore, Myo1b depletion or Arp2/3 inhibition could disrupt the interaction between the TGN membrane and F-actin, hampering secretory granule formation. These results suggest that the actomyosin complex could contribute to secretory granule biogenesis by triggering membrane deformation, which could be stabilized in the nascent secretory granule by cytosolic BAR proteins, such as the protein interacting with C kinase 1 or arfaptin-1^21, 22^, before the recruitment of scission proteins that will give rise to immature secretory granules.

The present work represents the first evidence that Myo1b and F-actin are involved in secretory granule formation and in the establishment of the regulated secretory pathway. The action of the actomyosin complex could require its recruitment at the TGN to induce membrane remodeling, a mandatory process for the biogenesis of secretory granules. Additional studies will be needed to establish the molecular mechanisms promoting the recruitment of actomyosin complex to the TGN membrane in order to trigger secretory granule biogenesis.

## Materials and Methods

### Cell culture

African green monkey kidney fibroblast-derived COS7 cells (American Type Culture Collection; CRL 1651), wild type (COS7-WT) and stably expressing CgA (COS7-CgA) developed previously^8^, were maintained in Dulbecco’s Modified Eagle’s Medium (DMEM, Sigma-Aldrich) supplemented with 5% heat-inactivated fetal bovine serum (Life Technologies), 100 U ml^−1^ penicillin and 100 µg ml^−1^ streptomycin (Life Technologies), and 300 µg ml^−1^ geneticin (G-418 sulfate, Life Technologies) to maintain selection in COS7-CgA cells, at 37 °C in 5% CO_2_. Rat pheochromocytoma PC12 cells (American Type Culture Collection; CRL 1721) were routinely grown in Dulbecco’s Modified Eagle’s Medium (DMEM, Sigma-Aldrich) supplemented with 5% sterile-filtered fetal bovine serum (Sigma-Aldrich), 10% sterile-filtered HyClone Donor Equine serum (Thermo Scientific), 100 U ml^−1^ penicillin and 100 µg ml^−1^ streptomycin (Life Technologies), 1% L-glutamine (Life Technologies), at 37 °C in 5% CO_2_. Most experiments with PC12 cells were performed on cells plated onto 10 µg ml^−1^ poly-D-lysine (Sigma) coated 15-mm round glass coverslips in 24-well plates. For IF and live experiments, COS7-WT cells were transfected with 0.8 μg of DNA encoding GFP-tagged human CgA (CgA-GFP)^8^ and 2 μl Lipofectamine 2000 (Invitrogen) per well (24-well plate or Matteck) according to the manufacturer’s protocol. Four or five hours after the beginning of transfection, the culture medium was replaced by supplemented DMEM, and cells were additionally cultured for 24–48 h for IF experiments.

### Myosin 1b knockdown

For transient Myo1b knockdown, a home-designed Myo1b siRNA (Myo1bH siRNA, 5′-GCTTACCTGGAAATCAACAAG-3′) (Sigma Proligo) was used as previously described^6^. A non-targeting siRNA pool designed by Dharmacon-Thermo Scientific has been used as control siRNA. The non-targeting pool contains 2 siRNA which respectively reduces EGFR mRNA by ≈50% and targets firefly luciferase. COS7 cells were transfected with 10 nM siRNA and 1 µL of Lipofectamine RNAimax (Life Technologies) per well, in 24-well plate, according to the manufacturer’s protocol. PC12 cells were transfected with 30 nM siRNA, following the same protocol. Four hours after the transfection, the medium was replaced by supplemented DMEM, and cells were cultured for 48 h. Transfected cells were then analyzed by SDS-PAGE and Western blotting for Myo1b or CgA levels or fixed for IF.

### Drug treatments

Arp2/3 complex was inactivated by incubating cells in DMEM supplemented with 100 µM DMSO-dissolved CK-666 (Sigma-Aldrich) for 1 h at 37 °C, 5% CO_2_. Actin filaments were depolymerized by incubating cells in DMEM supplemented with 2.5 µM latrunculin B (Calbiochem, La Jolla, CA) for 45 min at 37 C, 5% CO_2_. Under these conditions, microtubules were not significantly affected (data not shown).

### Secretion analysis

COS7 cells stably expressing CgA were cultured in 24-well plates, extensively washed with phosphate buffered saline and subsequently incubated in calcium secretion buffer (150 mM NaCl, 5 mM KCl, 2 mM CaCl2, 10 mM HEPES, pH 7.4) (basal release of CgA) or in barium secretion buffer (150 mM NaCl, 5 mM KCl, 2 mM BaCl2, 10 mM HEPES, pH 7.4) (stimulated release of CgA) for 15 min, at 37 °C in 5% CO_2_. Secretion media were collected, cleared by centrifugation (5 min, 14,000 *g*, 4 °C) and stored in Laemmli buffer for further analysis. Cells were lysed in NP40 cell lysis buffer (Invitrogen) containing protease inhibitor cocktail (Roche), and centrifuged (15 min, 15,000 *g*, 4 °C). Then, proteins from the supernatant were denatured in Laemmli buffer. Secretion media and cell homogenates were analyzed by Western blotting.

### Antibodies

Primary antibodies used were monoclonal anti-synaptobrevin 2 (104211 from Synaptic system; 1:5,000); monoclonal α-tubulin (T5168 from Sigma; 1:5,000); rabbit polyclonal anti-CgA (EL-35)^23^ (1:500 for IF, 1:1,000 for Western blotting); goat polyclonal anti-CgA (sc-23556 from Santa Cruz Biotechnology inc; 1: 200); rabbit polyclonal anti-Myo1b (HPA 013607 from Sigma prestige antibodies; 1:200 for IF, 1:250 for Western blotting); mouse monoclonal anti-GM130 (610822 from BDBiosciences; 1:1,000); rabbit polyclonal anti-furin (Ab3467 from Abcam; 1:200); sheep polyclonal anti-human TGN46 (AHP500 from AbD serotec; 1:500); rabbit polyclonal anti-p34-Arc/ARPC2 (07-227 from Millipore; 1:400); goat anti-type III collagen (1330-01 from Southern Biotech; 1: 200). For IF, secondary antibodies used were Alexa 488-conjugated donkey anti-rabbit IgG; Alexa 594-conjugated donkey anti-rabbit IgG; Alexa 647-conjugated donkey anti-rabbit IgG; Alexa 488-conjugated donkey anti-mouse IgG; Alexa 488-conjugated donkey anti-goat IgG; Alexa 594-conjugated donkey anti-goat IgG; Alexa 594-conjugated donkey anti-sheep IgG; Alexa 647-conjugated donkey anti-mouse IgG (Invitrogen; 1:500). For Western blotting, anti-rabbit, anti-mouse and anti-goat secondary antibodies conjugated to horseradish peroxydase (Santa Cruz biotechnologies; 1:2,000) were used.

### Protein electrophoresis and Western blotting

Cells were harvested by scraping, homogenized, and proteins were separated by SDS-PAGE followed by Western blotting. Membranes were incubated in a blocking buffer containing 5% non-fat dry milk in phosphate-buffered saline containing 0.05% Tween 20 (Sigma) (PBS-T) for 1 h at room temperature, and overnight with primary antibodies at 4 °C. Then, membranes were washed for 45 min with PBS-T. Blots were subsequently incubated for 1 h with appropriate HRP-conjugated secondary antibody in blocking buffer. Membranes were washed for 45 min with PBS-T. Immunoreactive proteins were detected by chemiluminescence (Pierce Biotechnology). Quantification was performed using ImageJ software (Wayne Rasband National Institutes of Health) and Image Lab software (Bio-Rad). The mean intensity of each individual band of interest was calculated after background value subtraction. For validation of siRNA transfection, values were normalized to the mean intensity of the loading control band for each sample (α-tubulin). For secretion studies, values of released CgA in the medium were normalized to those of total CgA (medium + cell content).

### Immunofluorescence labelling

Cells cultured onto coated glass coverslips were transfected as described above and fixed with 4% paraformaldehyde in PBS at room temperature for 15 min. Cells were permeabilized and blocked for 30 min with 0.3% Triton X-100 in PBS containing normal donkey serum (1:50) and 1% BSA. Cells were then incubated for 2 h at room temperature with primary antibodies, and, after washing with PBS, for 1 h with secondary antibodies. Phalloidin-FITC (Invitrogen, 500 nM) was used to detect actin filaments. Nuclei were stained with DAPI (Molecular probes #D3571, 1 µg ml^−1^). To verify the specificity of the immunoreactions, the primary or secondary antibodies were substituted with PBS.

### Image acquisition

Confocal microscopy was carried out with a TCS-SP8 upright confocal laser scanning microscope equipped with 63× oil immersion objective (NA = 1.4; Leica, Microsystems). Alexa 488 and GFP were excited at 488 nm and observed in a 505–540 nm window. Alexa 594 was excited at 594 nm and observed in a 600–630 nm window. Alexa 647 was excited at 633 nm and observed in a 650–700 nm window. For dual color acquisition, images were sequentially acquired in line scan mode (average line = 2). Overlays were performed with post acquisition Leica Confocal Software functions to obtain the presented snapshots. Golgi exit of granules was monitored by time-lapse fluorescence microscopy using a spinning-disc confocal microscope. This microscopy was carried out with a Yokogawa CSU-22 spinning-disc head on a Nikon TE-2000 U microscope equipped with a 100× NA 1.4 oil immersion objective and a Coolsnap HQ2 camera, a NanoScanZ piezo focusing stage (Prior Scientific) and a motorized scanning stage (Marzhauser). This microscope was steered with Metamorph 7.1 (Universal Imaging Corporation). The fast scan mode at 512 × 512 pixel resolution was used. Video sequences were acquired at 5 frames s^−1^ (100–200 ms exposures).

### Post-acquisition analysis

The extent of colocalization of two labels was measured using the “Colocalization” module of Imaris 7.6.5. 64-bit version (Bitplane AG, www.bitplane.com). This program analyzes plan of confocal sections acquired in two channels. Imaris colocalization analyzes the entire confocal plan by measuring the intensity of each label in each pixel. The program uses an iterative procedure to determine an intensity threshold (in the 0–255 scale of pixel intensity) for each of the two labels. Pixels with intensities above this threshold are considered to be above the background. A pixel is defined as having colocalization when the intensities of both labels are above their respective thresholds. The extent of colocalization was expressed by Pearson coefficient in pixels with colocalization. The Pearson coefficient is a number between + 1 and −1, with positive values indicating a direct correlation, negative values indicating an inverse correlation, and values near 0 indicating no correlation. In this case, the Pearson coefficient measures the correlation between the intensities of the two labels only in the pixels with colocalization. The following procedure was used to measure colocalization. A computer folder containing the stack of confocal sections for the two labels was generated by the Leica TCS-SP8 confocal microscope. This folder was opened with Imaris and converted into an Imaris file. A broad region of interest (ROI) was defined as all the pixels in which the intensity of one of the labels was above a pixel intensity defined in the 0–255 scale. Once the thresholds were set, the program outputs a canal of colocalization with channel statistics containing the Pearson coefficient. Then with the tool ‘spots detection’ on Imaris, the number of spots in each label (A, B and ‘coloc’ channel) was quantified to determine the percentage of colocalised granules. Spots statistics are automatically computed for each spot object. To each spot belongs a spatial position along the x- and y-axis, as well as the intensity of the point it represents. The spot object is available to model point-like structures in the data. It provides a procedure to automatically detect point-like structures, an editor to manually correct detection errors, a viewer to visualize the point-like structures as spheres, and statistics output. The rendering of the TGN surface was obtained using the Imaris ‘surface’ tool.

For the quantification of the number of CgA granules (Figs 2, 3, 5 and 6), the number of objects in the cytoplasm was counted automatically using the Imaris ‘spots’ function and a size filter (estimated xy diameter) of 0.5 µm. For the quantification of F-actin in the Golgi region, the Golgi region was first outlined on the plane showing more GM130 staining; then F-actin was thresholded and the overlapping pixels between the two structures was calculated with the Imaris ‘coloc’ function. The distributions of GM130 and F-actin were analysed using post acquisition ‘line scan’ mode of the Leica Confocal Software. For the analysis of live cell images, each granule in the Golgi area was identified after background correction by ND-Safir software^24^ (Inria Rennes). The mean velocity of detected granules was determined using the ImageJ “manual tracking” tool.

### Subcellular fractionation

Cells were collected in PBS and sedimented by centrifugation at 400 *g* for 5 min at 4 °C. The cell pellet was disrupted by 5 pull/push through a 21- and then a 25-gauge needles attached to a syringe, in ice-cold buffer (0.32 M sucrose, 20 mM Tris-HCl, pH 8; 1 ml g^−1^ of cells). The resulting lysate was centrifuged at 800 *g* for 30 min at 4 °C. Post-nuclear supernatants were centrifuged at 20,000 *g* for 20 min at 4 °C. Pellets containing dense core granules were centrifuged on a multi-step gradient of 1 to 2.2 M sucrose (1, 1.2, 1.4, 1.6, 1.8, 2 and 2.2 M sucrose; 5 ml steps), at 100,000 *g* for 12 h at 4 °C. All gradient steps were collected from the top of the tube in 5 ml fractions, and analyzed by western blotting to check the granule-containing fractions and to verify their purity. The recovered granule fractions were used for liquid chromatography coupled to tandem mass spectrometry analysis.

### Proteomic analysis

The fraction containing CgA granules purified from COS7-CgA cells was diluted in Buffer A (20 mM Tris pH 7.5, 150 mM NaCl and Complete Inhibitors (Roche)). The sample was submitted to two freeze-thaw cycles, sonicated for 1 min in a cold water bath and centrifuged at 50,000 *g* for 1.5 h at 4 °C to separate soluble from insoluble material. In order to precipitate soluble proteins, the soluble fraction was diluted in 4 volumes of cold (−20 °C) acetone and incubated for 4 h at −20 °C before centrifugation at 13,000 *g* for protein precipitation. On the other hand, the pellet containing the insoluble material was carefully washed twice with cold buffer A and incubated in 200 µl of Buffer A containing 1% n-dodecyl-beta-maltoside and 0.4% amidosulfobetaine-14 detergents in order to efficiently solubilize membrane proteins. After 2 h of incubation under soft agitation at 4 °C, the extract was centrifuged for 10 min at 13,000 *g* and the proteins contained in the supernatant were precipitated using standard chloroform/methanol method. Then, proteins were reduced, alkylated and separated by 1D- gel electrophoresis. Finally, proteins were cleaved in gel by trypsin and identified by liquid chromatography coupled to tandem mass spectrometry.

### Statistical analysis

Data were analyzed with the Prism program (GraphPad Software). All secretion experiments were repeated at least four times using the non-parametric Mann-Whitney U test. For the quantification of the number of CgA granules, statistical significance was determined by Student’s *t* –test with Welch’s correction or Mann-Whitney U test. Values are expressed as means ± s.e.m., and the level of significance is designated in the figure legend as follows: *P < 0.05, **P < 0.01, ***P < 0.001, ****P < 0.0001.

## Electronic supplementary material

supplementary info

movie S1

movie S2
