# Supplementary material for: Myosin 1b and F-actin are involved in the control of secretory granule biogenesis
Source: Sci Rep. 2017 Jul 12;7:5172. doi: 10.1038/s41598-017-05617-1 (PMC5507975; doi:10.1038/s41598-017-05617-1)
Supplement: Supplementary file 1 — supplementary info [file 41598_2017_5617_MOESM1_ESM.pdf]

Supplementary information for

Myosin 1b and F-actin are involved in the control of secretory granule biogenesis

Charlène Delestre-Delacour[1,+], Ophélie Carmon[1,+], Fanny Laguerre[1], Catherine Estay-Ahumada[2], Maïté Courel[1,$], Salah Elias[1,£], Lydie Jeandel[1], Margarita Villar Rayo[3], Juan R. Peinado[4], Lucie Sengmanivong[5], Stéphane Gasman[2], Evelyne Coudrier[6], Youssef Anouar[1,*], and Maité Montero-Hadjadje[1,*]

This file includes:

1. Supplementary Figure S1
2. Supplementary Figure S2
3. Legend of supplementary Movie S1
4. Legend of supplementary Movie S2

Supplementary Figure S1. Full image of the nitrocellulose sheet that has been used to generate panel A of Figure 1. Two subcellular fractionations (secretory granules 1 and 2) of PC12 cells have been performed and proteins were separated on SDS-PAGE and blotted on nitrocellulose. The nitrocellulose sheet has been sliced in three pieces as indicated in order to estimate the levels of Myo1b and VAMP2 in the same fraction. Western blots were performed by chemiluminescence using the Super Signal West Dura Extended Duration Substrate system (Pierce). Immunoreactive bands were detected using the image acquisition system Chemi-smart 5000 generating original images as tiff files. Lanes 1 (PC12 cell lysate) and 5 (PC12 secretory granules) have been used to generate Figure 1A (white squares indicated the cropped images).

Supplementary Figure S2. Full scan of blots that have been cropped in panels A, B and C of Figure 2. Black squares indicated the cropped images.

Supplementary Movie S1. Dynamics of CgA granules in mock conditions. COS7 cells were transfected with control siRNA and with a plasmid encoding CgA-GFP. CgA-GFP carriers were monitored 5 h after cell transfection at 37°C by time-lapse imaging using spinning-disc confocal microscopy.

Supplementary Movie S2. Dynamics of CgA granules after Myo1b depletion. COS7 cells were transfected with Myo1b siRNA and with a plasmid encoding CgA-GFP. CgA-GFP carriers were monitored 5 h after cell transfection at 37°C by time-lapse imaging using spinning-disc confocal microscopy.

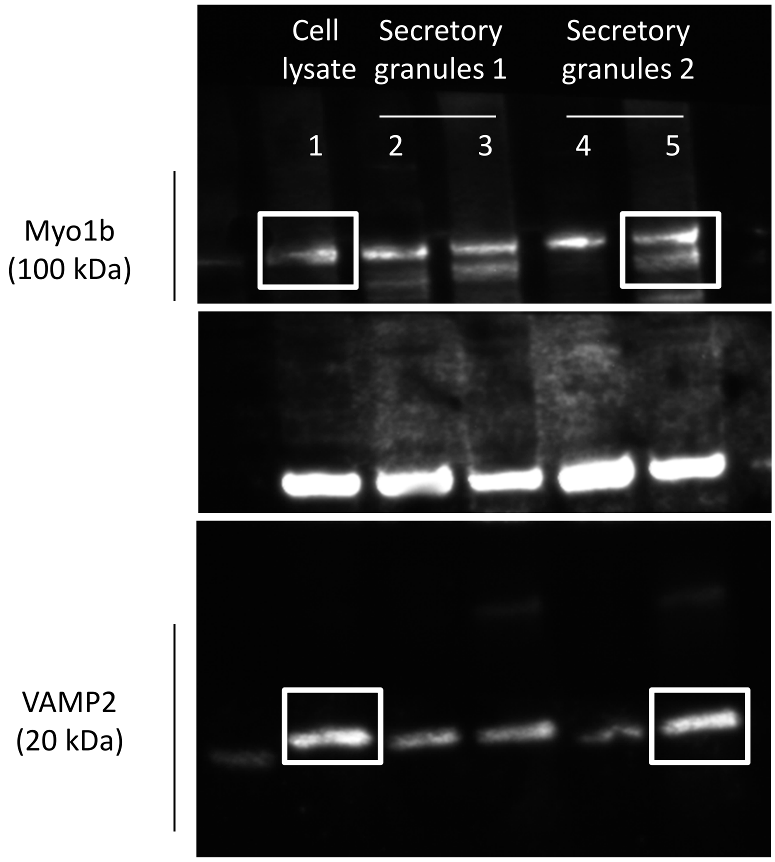
Cell lysate
Secretory granules 1
Secretory granules 2
1
2
3
4
5
Myo1b (100 kDa)
VAMP2 (20 kDa)

Figure S1

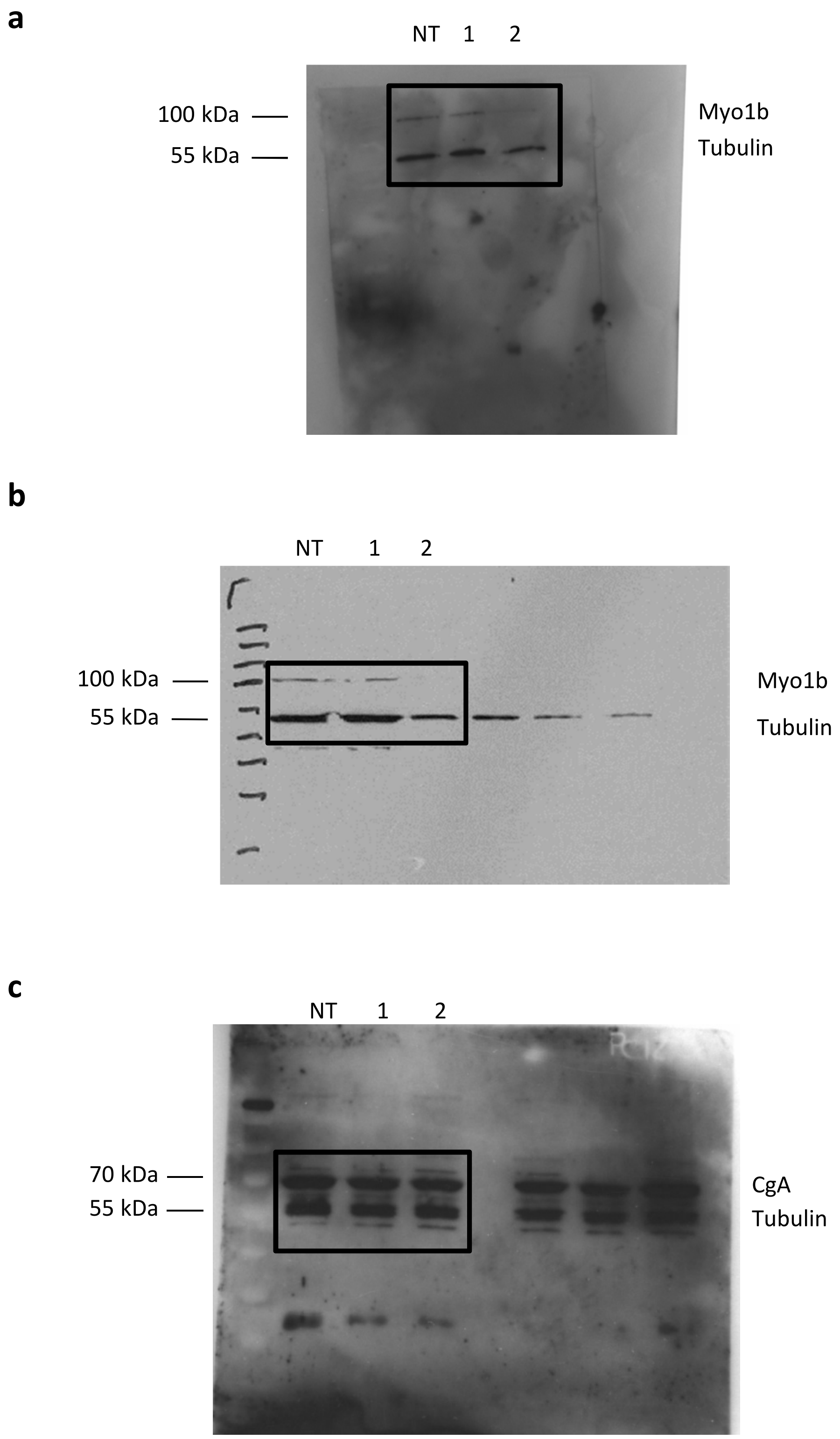
a
NT 1 2
100 kDa
55 kDa
Myo1b
Tubulin
b
NT 1 2
100 kDa
55 kDa
Myo1b
Tubulin
c
NT 1 2
70 kDa
55 kDa
CgA
Tubulin

Figure S2
